# Hajdu Cheney syndrome; A novel NOTCH2 mutation in a Syrian child, and treatment with zolidronic acid: A case report and a literature review of treatments

**DOI:** 10.1016/j.amsu.2021.103023

**Published:** 2021-11-03

**Authors:** Afaf Ahmad, Haya Deeb, Diana Alasmar

**Affiliations:** a Faculty of Medicine, Damascus University, Damascus, Syria; b Children University Hospital, Damascus, Syria

**Keywords:** Hajdu-Cheney syndrome, NOTCH2 mutation, Osteoporosis, Bisphosphonates, Zolidronic acid

## Abstract

**Introduction:**

Hajdu Cheney Syndrome (HCS) is a rare genetic disorder characterized by skeletal deformities such as acroosteolysis, osteoporosis, unique craniofacial features, and other systemic abnormalities. This syndrome is caused by NOTCH2 gene mutations, which cause an increase of osteoclast and osteoblast activity that leads to the increased bone resorption. Because of how rare the syndrome is and the vague onset of the symptoms, it can be challenging to make an early diagnosis.

**Case presentation:**

We report a case of a female child with HCS who has a new NOTCH2 mutation sequence; (NM_024408.3:c.6463G > T) protein change (Glu2155*), and to our knowledge this is the first reported and diagnosed case in Syria. She presents with short stature, unique craniofacial features, scoliosis, kyphosis, and signs of osteoporosis, in addition to Patent Ductus Arteriosus. The patient was diagnosed with Hajdu Cheney Syndrome, and administered zolidronic acid, and she responded well to the treatment; showing signs of improved bone density and improvement in height, where her bone density improved from 0.23 to 0.31, and she gained 11 cm in height after the treatment.

**Conclusion:**

Due to the rarity of the syndrome, there is no established guideline for treatment yet. Based on the pathophysiology of the syndrome that causes increased bone resorption, treatment with the Bisphosphonates group has yielded positive outcomes. Furthermore, we compare different treatments in the literature with their results.

## Highlights

• Hajdu Cheney Syndrome is caused by rare genetic disorders involving the NOTCH-2 gene.
• It presents with skeletal deformities, osteoporosis and unique facial features.
• Whole Exome Sequencing is the best diagnostic tool to confirm the diagnosis.
• We report a novel NOTCH2 Mutation: (NM_024408.3:c.6463G > T) protein change (Glu2155*).
• Treatment with Bisphosphonates has shown significant improvement in the patient.

## Abbreviations

HCS: Hajdu Cheney Syndrome
PDA: Patent Ductus Arteriosus
DXA: dual energy X-ray absorptiometry
BMD: Bone Mineral Density
MPS: Mucopolysaccaride disorders
WES: Whole Exome Sequencing
FPPS: farnesyl pyrophosphate synthase
IV: Intra-venous

## 1 Introduction

Hajdu-Cheney Syndrome (HCS), first described by Hajdu in 1948 and reported as a syndrome by Cheney in 1962 [1], is a rare genetic disorder characterized by acroosteolysis, severe osteoporosis with frequent fractures, short stature, and craniofacial developmental defects such as platybasia, open sutures, and wormian bones. Additionally, patients usually present with cardiovascular septal and valvular defects, neurological complications, and renal cysts [2,3].

HCS is a dominantly inherited genetic disorder, although most reported cases are sporadic. HCS is associated with mutations in exon 34 of the NOTCH2 gene [4,5]**.** Most mutations are either nonsense mutations or deletion mutations, both leading to the formation of a termination codon upstream of the PEST domain, which is essential to the degradation of NOTCH2. Thus, the mutation leads to the expression of a NOTCH2 truncated protein, causing a gain of function to NOTCH2. These mutations are associated with the clinical manifestations observed in HCS [2].

Due to the rarity of the disease, with a prevalence of 1 person for every 1 million, there is no established treatment guidelines in place yet [6].

In this report, we will be presenting a case report on a female child diagnosed with HCS, which marks the first reported and diagnosed case of HCS in Syria. Additionally, we will present a brief literature review of the attempted treatments available thus afar in the literature with their results.

This case report has been reported in line with the SCARE Criteria [35].

## 2 Case presentation

**Fig. 1:**
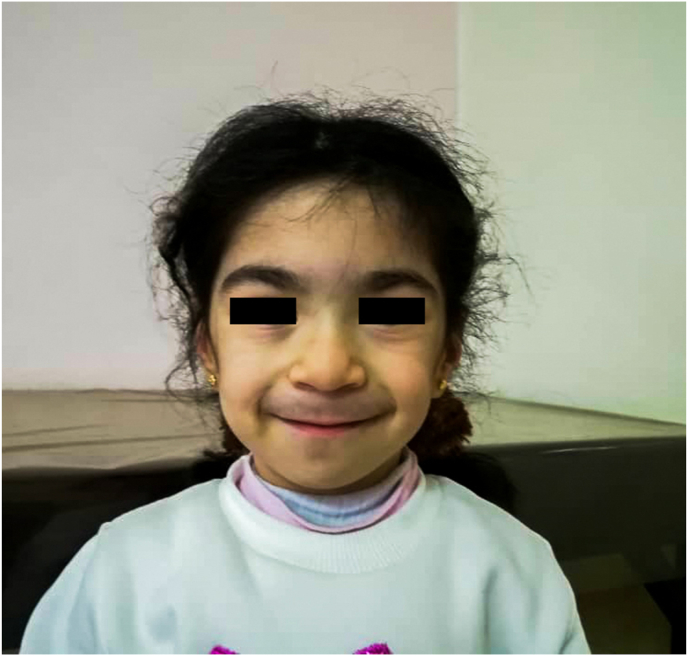
The craniofacial features that show flat nasal bridge, low set ears, hirsutism, telecanthus, and other features.

**Fig. 2:**
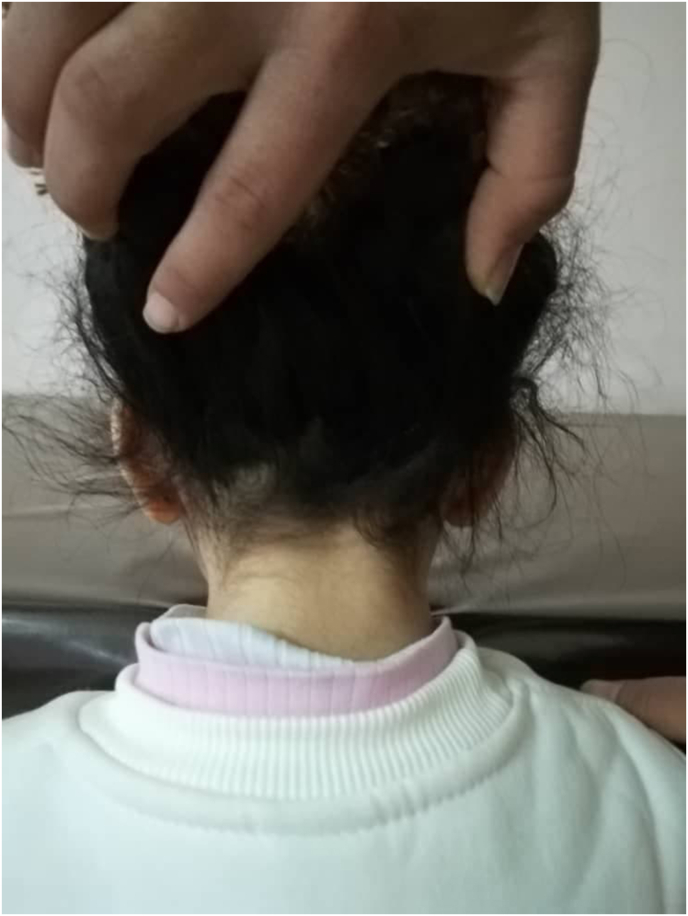
A picture of the patient's neck, showcasing the short neck the patient has.

In 2019, a 5-year-old girl was referred to the metabolism department in the Children's University Hospital in Damascus with a complaint of short stature, with a height of 96 cm and a weight of 13 kg. She presented with developmental delay, stubby fingers, and unique facial features that included: coarse face, facial hirsutism, bushy eyebrows, telecanthus, flat nasal bridge, long philtrum, low set ears, and a short neck (Fig. 1, Fig. 2). The patient had a history of jerking seizures, and was treated with Phenobarbital and Phenytoin. Additionally, the patient had Patent Ductus Arteriosus (PDA) with normal pulmonary pressure, for which she was prescribed Digoxin.

**Fig. 3:**
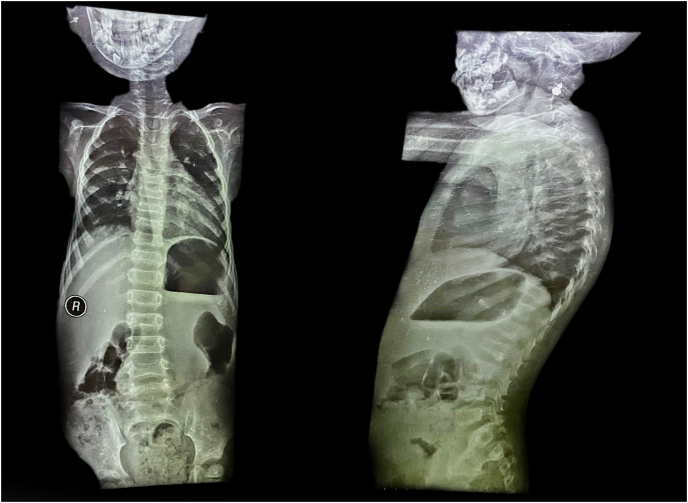
A spine X-ray in the lateral position and anterior-posterior position showing the scoliosis and kyphosis that the patient has.

**Fig. 4:**
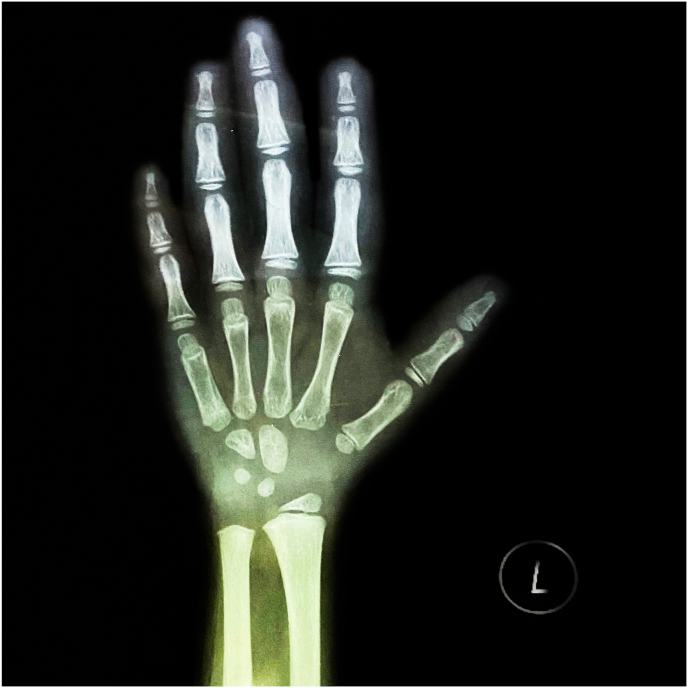
Left hand x-ray showing mild acroosteolysis in the distal phalanges.

**Fig. 5:**
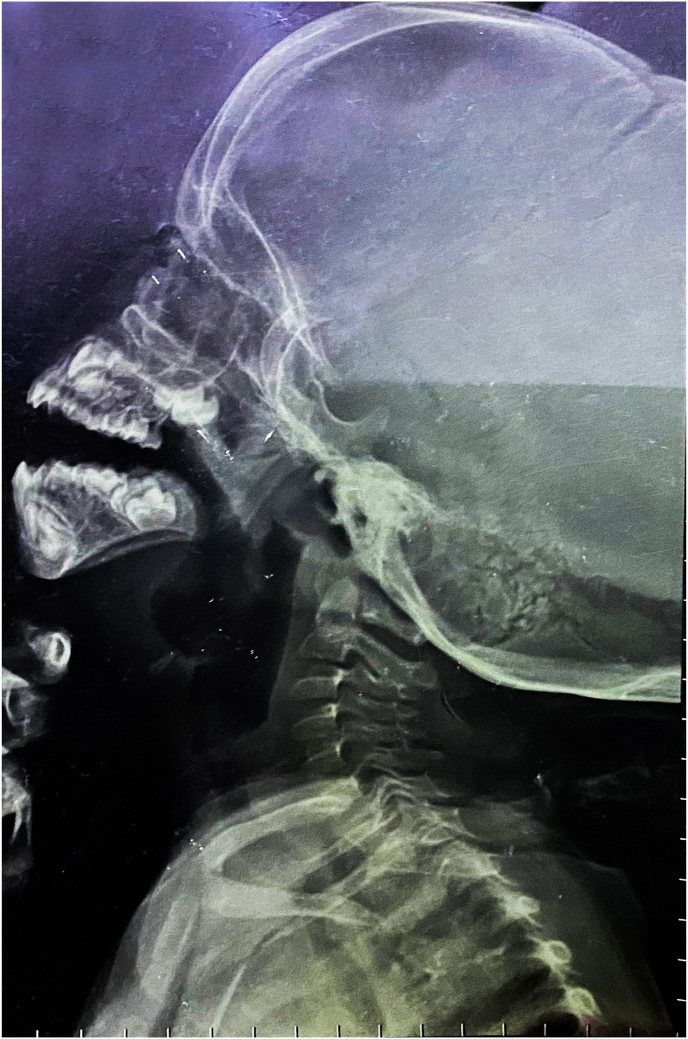
Head and neck x-ray showing elongated sella turcica and an elevated palate.

For further investigation, radiographs and laboratory tests were performed. Spine radiographs showed mild kyphoscoliosis, and signs of osteoporosis in the form of reduction of vertebral body height (Fig. 3), which also had low density on the radiographs. Left hand radiographs showed mild acroosteolysis in the distal phalanges, and due to the early onset of the symptoms and the early diagnosis, the bone lysis is not severe and frank (Fig. 4). Head and neck radiographs showed that the patient had elongated sella turcica and an elevated palate (Fig. 5). Bone densitometry with dual energy X-ray absorptiometry (DXA) was performed and the results showed a lumbar spine Z-score of −4.8 (−46%) and a bone mineral density (BMD) of 0.232 for the lumbar vertebras. Laboratory results and the karyotype were normal.

Osteogenesis Imperfecta was suspected, but the clinical and facial features pointed towards mucopolysaccaride disorders (MPS), which were excluded as a definitive diagnosis after showing normal results for the screening of lysosomal MPS enzymes. This warranted a whole exome sequencing (WES) to screen for other possible genetic disorders. WES results came positive for autosomal dominant NOTCH2 gene-related disorders. The sequencing revealed a heterozygous nonsense mutation (NM_024408.3:c.6463G > T) protein change (Glu2155*), which creates a premature stop codon.

**Fig. 6:**
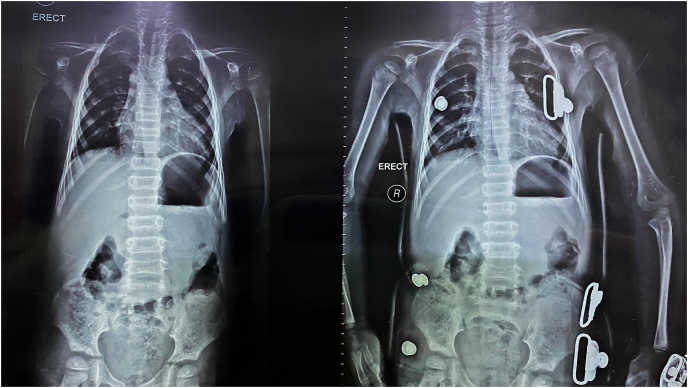
A spine X-ray in the anterior-posterior position showing the improvement in the spine deformities after using a back brace.

Pairing the genetic results with the clinical features, the patient was diagnosed with Hajdu-Cheney Syndrome in February 2020, and was started on 0.05 mg/kg/dose of IV zolidronic acid every six months, in addition to calcium and vitamin D supplements due to very low levels of vitamin D, and the patient was prescribed a back brace to correct the spine deformities (see Fig. 6).

On the first follow up in October 2020, the patient's height and laboratory results had improved. Ophthalmological exam was performed, showing a mild cloudy cornea, retina hypoplasia, normal ocular pressure and optic nerve features. Vision acuity was normal (10/10).

The patient's second follow up in April 2021 showed significant improvement in the patient's height, BMD and Z-score (Table 1). The patient's Z-score, which was −4.8 at the time of the diagnosis, improved to −3.3 after the administration of Zoledronic Acid. Additionally, her BMD improved from 0.23 to 0.31, and she gained 11 cm in height over the course of the 2 years follow up.

**Table 1:** Assessment of the patient's height, weight, Z-score, BMD, Vitamin D and Calcium levels.

Date	June 2019	February 2020 ∗	April 2020	October 2020	March 2021
Calcium (mg/dl)	9.7	Nd	9	9.2	9.4
Vitamin D (ng/ml)	40	10	18.8	28.8	56
Height (cm)	95	–	–	103	106
Weight (kg)	13	–	–	15.5	17.5
Z-score	−4.8 (−46%)	–	–	–	−3.3 (−36%)
BMD	0.232	–	–	–	0.310

∗ Patient was started on Zolidronic Acid.

## 3 Discussion

Hajdu Cheney Syndrome (HCS) is a rare genetic disorder that classically presents with short stature, acroosteolysis, unique facial features that include having a coarse face, flat nasal bridge, facial hirsutism, micrognathia, telecanthus, long philtrum, low set ears, and other features. Additionally, it presents with significant skeletal features like kyphosis and scoliosis. Other systemic findings include renal, cardiac, neurological, visual, auditory and intestinal abnormalities [[7], [8], [9], [10]]**.** The case of this report presented with the classical facial features of HCS, in addition to presenting with short stature, scoliosis, kyphosis, and PDA. However, the patient did not present with any renal or intestinal or auditory abnormalities, but she did have visual problems such as cloudy cornea and retinal hypoplasia.

There are distinct radiological findings due to the osteolysis and osteoporosis, like acroosteolysis of the distal phalanges of the hands and feet, open sutures, abnormal flattening of the skull, elongated sella turcica, and loss of spinal bone density. These findings can be detected using radiographs and calculating the BMD [[11], [12], [13]]. The patient in our case has similar radiological features as the ones associated with HCS. These radiological and clinical features are common with diseases such as osteogenesis imperfecta, mucopolysaccaride disorders, and HCS. Whole exome sequencing is always required as a genetic test to make the definitive diagnosis.

NOTCH2 is a transmembrane receptor [14] that plays an important role in bone homeostasis, development of the skeleton, and in bone remodeling by acting on the osteoclasts and osteoblasts lineage [2,[15], [16], [17], [18]]. The genetic mutation associated with HCS is a nonsense or deletion mutation that creates a termination codon in exon 34 of NOTCH2 upstream the PEST domain [5,11,[19], [20], [21]]. This mutation gives rise to the craniofacial and skeletal features in this syndrome. In our reported case, the patient had a heterozygous nonsense NOTCH2 mutation with the sequence (NM_024408.3:c.6463G > T) p. (Glu2155*). This particular sequence, to our knowledge, has not been reported in previous case reports nor described in the literature [[22], [23], [24]]**.**

Due to the pathophysiology of the syndrome, which causes an increase of osteoclast and osteoblast activity that leads to the increased bone resorption [11,25,26], managing this syndrome requires an approach that tackles these particular effects. The drug that is most commonly used to treat osteoporosis is the bisphosphonate class [27]. Bisphosphonate has a high affinity for bone minerals, which achieves a high local concentration in the skeleton, making it the most effective drug in disorders with skeletal remodeling imbalance (like HCS), which lead to excessive osteoclast-related bone resorption [28]. Nitrogen containing bisphosphonate sub-class effectively inhibits the activity of farnesyl pyrophosphate synthase (FPPS), a key regulatory enzyme in the mevalonic acid pathway, which is responsible for the synthesis of cholesterol [29,30]**.**

Compared with other nitrogen-containing bisphosphonates, zoledronate has the highest affinity for bone mineral and to inhibiting FPPS [31]**.** In vitro and in vivo clinical studies have shown that the order of efficacy in the inhibition of FPPS and bone resorption (from highest to lowest) is: zoledronate > risedronate > ibandronate > alendronate [27,29]. Based on these studies, we concluded that the use of zolidronic acid would be the most fitting course of treatment for our patient, and it showed significant improvement in the patient's BMD.

Due to the rarity of HCS, not enough literature is provided to study and conclude a treatment guideline. Provided below is a literature review that sums up the treatment regimens used in available literature to treat HCS (Table 2).

### 3.1 Literature review of the treatment

**Table 2:** Literature Review of the Treatments

Article	Patient	Age (years old)	Drug	Dose	Route of drug	Period of treatment	Z-Score baseline	1 year of Follow up	2 years of follow up
Sakka et al. [32]	P1	15	Zolidronic Acid	0.05 mg/kg/dose	IV 1 dose/6 months	2 years	−3.3	−2.4	−2.4
	P2	6.8	Pamidronate	1 mg/kg/dose	IV 1 dose/3 months	1 year	−1.8	−1.2	–
		7.8	Alendronate	35 mg	Oral weekly	3 years	−1.2	−0.9	–
		10.8	Alendronate	70 mg	Oral weekly	2.5 years	−0.3	−0.05	0.2
		17.5	Zolidronic Acid	5 mg	One IV infusion	–	−2	−1.6	–
	P3	15.5	Zolidronic Acid	0.05 mg/kg/dose	IV 1 dose/6 months	1.5 years	−0.3	−0.7	−1
	P4	10	Alendronate	5 mg	Oral daily	3 years	−0.5	0.4	1.1
		13	Alendronate	35 mg	Oral weekly	2 years	1.9	2.8	2
		15	Alendronate	70 mg	Oral weekly	5 years	2	1.5	0.9
		22	Alendronate	70 mg	Oral weekly	2 years	0.4	0.3	0.4
Adami et al.^a^ [4]	P5	33	Denosumab^1^	60 mg	Subcutaneously 1 dose/6 months	4 years	Lumber: −2.9	T: -3	−2.7
							Femoral neck: - 0.1	T: -1.2	−1.2
Esfstathiadou et al. [33]	P6	48	Zolidronic Acid	5 mg	IV 1 dose/1 year	2 years	T: -4	−3.7	−3.5
	P7	18	Zolidronic Acid^2^	5 mg	IV 1 dose/1 year	1 years and 4 months	−3.4	–	–
	P8	28	Zolidronic Acid	5 mg	IV 1 dose/1 year	2 years	−4.1	−4.1	−4.1
Pittaway et al.^b^ [34]	P9	6	Pamidronate	3 mg/kg/dose	IV 1 dose/3 months	1.5 years	−3.1	−1.3 (after 6 years)	
	P10	8	Pamidronate	3 mg/kg/dose	IV 1 dose/4 months	1.5 years	−1.7	−0.3 (after 1.5years)	
		24	Zolidronic Acid	5 mg	One infusion	1 years	−2.9	−2.5 (after 2.5 years)	
	P11	11	Alendronate	35 mg	Oral weekly	2 years	−5.6	−2.6 (after 5 years)	
			Alendronate	70 mg	Oral weekly	3 years			
	P12	15	Pamidronate	3 mg/kg/dose	IV 1 dose/3 months	3 years	−4.4	−4.5 (after 3 years)	
	P13	35	Zolidronic Acid	5 mg	IV 1 dose/1 year	4 years	−3.2	−2.8 (after 4 years)	
	P14	36	Alendronate	10 mg	Oral weekly	6 years	−3.0	−4.5 (after 6 years)	
	P15	39	Pamidronate	30–60 mg	IV 1 dose/3 months	8 years	−3.6	−3.0 (after 8.5 years)	
			Zolidronic Acid	4–5 mg	IV 1 dose/6 months	5 years			

a t score was used as an evaluation tool in the follow ups.

b the follow up period is inconsistent for the patients in this study.

## 4 Conclusion

Whole exome sequencing is a recommended tool to rule out other disorders and to confirm HCS, because the syndrome is rare and is rarely identified as a possible differential diagnosis. The patient's genome sequence (NM_024408.3:c.6463G > T) protein change (Glu2155*) is a novel sequence that has never been reported before in literature. Based on its pathophysiology, treatment with bisphosphonate group is recommended and it has shown good results. More studies are needed to establish a treatment guideline for this syndrome.

## Funding

There were no sources of funding.

## Ethical approval

No ethical approval was needed.

## Sources of funding

There were no sources of funding.

## Authors contribution

Afaf Ahmad: wrote the introduction, case presentation, the discussion, captions of the figures, provided the language-editing services, and reviewed the literature. Haya Deeb: wrote the abstract, discussion, provided the literature review, designed the figures, and reviewed the literature. Diana Alasmar: supervised the scientific and academic aspects of the manuscript preparation and submission, Hajdu Cheney Syndrome; a Novel NOTCH2 Mutation, and Treatment with Zolidronic Acid: A Case Report and a Literature Review of Treatments.

## Patient's consent

Written informed consent was obtained from the patient for publication of this case report and accompanying images. A copy of the written consent is available for review by the Editor-in-Chief of this journal on request.

## Registration of Research Studies

Name of the registry: This paper does not contain any research involving human participants.

Unique Identifying number or registration ID:

Hyperlink to your specific registration (must be publicly accessible and will be checked):

## Guarantor

Afaf Ahmad.

## Provenance and peer review

Not commissioned, externally peer-reviewed.

## Availability of data and materials

All data are available from the corresponding author on reasonable request.

## Declaration of competing interest

All the authors declared that they have no conflicts of interest.
