# Supplementary material for: Hajdu Cheney syndrome; A novel NOTCH2 mutation in a Syrian child, and treatment with zolidronic acid: A case report and a literature review of treatments
Source: Ann Med Surg (Lond). 2021 Nov 3;71:103023. doi: 10.1016/j.amsu.2021.103023 (PMC8606845; doi:10.1016/j.amsu.2021.103023)
Supplement: Multimedia component 2 [file mmc2.docx]

| Topic | Item | Checklist item description | Reported on line |
| --- | --- | --- | --- |
| **Title** | **1** | The diagnosis or intervention of primary focus followed by the words “case report” | 1 |
| **Keywords** | **2** | 2 to 5 key words that identify diagnoses or interventions in this case report, including "case report" | 25 |
| **Abstract** | **3a** | Introduction: What is unique about this case and what does it add to the scientific literature? | 12 |
|  | **3b** | Main symptoms and/or important clinical findings | 17 |
|  | **3c** | The main diagnoses, therapeutic interventions, and outcomes | 21 |
|  | **3d** | Conclusion—What is the main “take-away” lesson(s) from this case? | 24 |
| **Introduction** | **4** | One or two paragraphs summarizing why this case is unique (may include references) | 39 |
| **Patient Information** | **5a** | De-identified patient specific information | 56 |
|  | **5b** | Primary concerns and symptoms of the patient | 57 |
|  | **5c** | Medical, family, and psycho-social history including relevant genetic information | 62, 76 |
|  | **5d** | Relevant past interventions with outcomes | 80 |
| **Clinical Findings** | **6** | Describe significant physical examination (PE) and important clinical findings | 65 |
| **Timeline** | **7** | Historical and current information from this episode of care organized as a timeline | 58, 65, 70, 75, 76,79, 80 |
| **Diagnostic Assessment** | **8a** | Diagnostic testing (such as PE, laboratory testing, imaging, surveys) | 65-76 |
|  | **8b** | Diagnostic challenges (such as access to testing, financial, or cultural) | - |
|  | **8c** | Diagnosis (including other diagnoses considered) | 73 |
|  | **8d** | Prognosis (such as staging in oncology) where applicable | - |
| **Therapeutic Intervention** | **9a** | Types of therapeutic intervention (such as pharmacologic, surgical, preventive, self-care) | 80-82 |
|  | **9b** | Administration of therapeutic intervention (such as dosage, strength, duration) | 80 |
|  | **9c** | Changes in therapeutic intervention (with rationale) | - |
| **Follow-up and Outcomes** | **10a** | Clinician and patient-assessed outcomes (if available) | Not available |
|  | **10b** | Important follow-up diagnostic and other test results | 83-90 |
|  | **10c** | Intervention adherence and tolerability (How was this assessed?) | 83-90 |
|  | **10d** | Adverse and unanticipated events | No adverse effects |
| **Discussion** | **11a** | A scientific discussion of the strengths AND limitations associated with this case report | 130-132 |
|  | **11b** | Discussion of the relevant medical literature **with references** | 91-134 |
|  | **11c** | The scientific rationale for any conclusions (including assessment of possible causes) | 115 |
|  | **11d** | The primary “take-away” lessons of this case report (without references) in a one paragraph conclusion | 135 |
| **Patient Perspective** | **12** | The patient should share their perspective in one to two paragraphs on the treatment(s) they received | Not applicable |
| **Informed Consent** | **13** | Did the patient give informed consent? Please provide if requested | 147 |
